# Differential burden of diabetic foot ulcers in individuals with type 1 and type 2 diabetes: A narrative review of prevalence, healing, recurrence, and outcomes

**DOI:** 10.1177/00368504261465748

**Published:** 2026-07-13

**Authors:** Dimitrios Kyriazis, Anastasios Tentolouris, Ioanna Eleftheriadou, Konstantinos Manganas, Evangelia Tzeravini, Nikolaos Tentolouris

**Affiliations:** 1 First Department of Propaedeutic Internal Medicine and Diabetes Center, School of Medicine, 68993 National and Kapodistrian University of Athens, Laiko General Hospital, Athens, Greece; 2 Fourth Department of Internal Medicine, 69032 Evangelismos General Hospital, Athens, Greece

**Keywords:** diabetic foot ulcers, type 1 diabetes, type 2 diabetes, wound healing, lower-extremity amputation, diabetic foot infection

## Abstract

Diabetic foot ulcers (DFUs) are a major cause of morbidity and mortality in people with diabetes. Understanding differences in DFU burden and outcomes between T1DM and T2DM is important for advancing precision medicine. This narrative review summarizes evidence reporting DFU prevalence, healing, recurrence, infection, and lower extremity amputation outcomes separately in T1DM and T2DM. Population surveys suggest a higher lifetime prevalence in T1DM, driven by longer disease duration, whereas registry-based and database studies generally report higher annual incidence rates in T2DM, reflecting older age and macrovascular comorbidities. Crucially, most included studies lacked the multivariable adjustment and statistical power necessary to isolate diabetes type as an independent prognostic driver. Consequently, a lack of statistically significant differences should not be misinterpreted as proof of clinical equivalence. Overall, the available evidence suggests that time to healing does not differ substantially between individuals with T1DM and T2DM, though outcomes were primarily driven by local wound characteristics and vascular status. Recurrence is frequent and remains a clinical challenge irrespective of diabetes type, although people with T2DM may experience a greater burden. Evidence further indicates that the risk of LEAs is increased in both T1DM and T2DM, although the magnitude and comparative contribution of each diabetes type varies across populations and study designs, with real-world clinical cohorts indicating that advanced age and a neuro-ischemic phenotype in T2DM drive substantial post-ulcer morbidity. Finally, a higher relative risk of deep bone infections (osteomyelitis) emerges as one of the most severe diabetes-related foot infections and is associated with poor outcomes, with several reports suggesting a particularly pronounced burden among people with T1DM. Differences in DFU burden and outcomes between T1DM and T2DM appear to be driven more by patient phenotype than diabetes type itself. Precision-medicine approaches may help improve future prevention and management strategies.

## 1. Introduction

Diabetic foot ulcers (DFUs) represent one of the most serious complications of diabetes, with lifetime occurrence estimates ranging from roughly 19% to 34%.^1,2^ In addition to the substantial morbidity, DFUs are associated with markedly increased mortality; only 50–60% of affected individuals survive five years after ulcer onset.^3^ Between 5% and 24% of ulcers progress to amputation within 6–18 months of initial presentation, contributing to a 10- to 20-fold higher rate of non-traumatic lower-extremity amputations (LEA) in people with diabetes.^1,2^ DFUs also impose a considerable physical and psychological burden on patients by impairing mobility, increasing healthcare utilization, and diminishing overall quality of life.^4^

In recent years, a range of new pharmacological and non-pharmacological therapies has emerged for the management of DFUs.^5–8^ However, as clinical care moves increasingly toward personalized medicine rather than a “one-size-fits-all” approach, it is important to understand whether individuals with type 1 diabetes (T1DM) and type 2 diabetes (T2DM) differ in terms of DFU prevalence, healing rates, and complication profiles and whether such differences are better explained by underlying clinical phenotypes rather than diabetes type per se. Identifying such differences may help determine whether tailored therapeutic strategies are warranted for each diabetes type.

Importantly, emerging evidence suggests that diabetes type per se may not independently determine DFU prognosis, but rather reflects distinct clinical phenotypes characterized by differences in age, diabetes duration, metabolic profile, neuropathy, and peripheral arterial disease, which in turn influence ulcer presentation and outcomes. Accordingly, understanding these differences may help determine whether tailored preventive or therapeutic strategies are warranted based on clinical phenotype and risk profile rather than diabetes type alone.

The aim of this narrative review was to examine differences between T1DM and T2DM in the prevalence of DFUs, ulcer course and healing outcomes, and major complications, including LEAs and infection-related events, with particular emphasis on whether such differences reflect underlying clinical phenotypes rather than diabetes type itself.

## 2. Methods

This narrative review was guided by the Scale for the Assessment of Narrative Review Articles (SANRA) framework to ensure structural and reporting quality.^9^ This manuscript synthesizes evidence from population-based cohorts, national registries, and specialist clinical studies reporting DFU prevalence, healing outcomes, recurrence, infections, and lower extremity amputations stratified by diabetes type (T1DM vs T2DM). A structured literature search was conducted in PubMed/MEDLINE using combinations of relevant keywords (e.g. “diabetic foot ulcer”, “type 1 diabetes”, “type 2 diabetes”, “wound healing”, “recurrence”, “diabetic foot infection”, “lower-extremity amputation”). Studies were selected based on reporting DFU-related outcomes stratified by diabetes type. We specifically included population-based cohorts, registry studies, clinical observational studies, and comparative studies that provided sufficient data granularity for descriptive comparison, while small case reports or studies lacking clear clinical definitions were excluded. Potential selection biases in this narrative review include the restriction to English-language publications indexed in PubMed/MEDLINE, which may omit regional registry data, the exclusion of non-English publications, the inherent over-representation of high-quality data from well-established national registries (e.g. UK databases), potentially limiting global generalizability, and the lack of a formal, standardized risk-of-bias assessment for individual studies. Due to the narrative design of the review, no formal systematic review methodology or meta-analysis was performed, and results were synthesized descriptively. Across the included studies, statistical approaches were heterogeneous and adjustment for multiple testing was inconsistently reported. Results were therefore synthesized descriptively without formal correction for multiple comparisons. To explore whether DFU outcomes might reflect underlying clinical phenotypes rather than diabetes type per se, our descriptive synthesis utilized a comparative qualitative approach. For each included study, we evaluated: (i) the baseline clinical and demographic characteristics of the T1DM and T2DM cohorts (e.g., disparities in age, disease duration, and peripheral arterial disease prevalence), and (ii) whether associations between diabetes type and outcomes remained significant after multivariable adjustment for these clinical factors. This approach allowed us to interpret differences in outcomes in relation to underlying clinical characteristics, comorbidity burden, and study setting, rather than attributing findings solely to diabetes type itself.

## 3. Prevalence of diabetic foot ulcers

In this section, we summarize evidence from retrospective and cross-sectional studies that have quantified the prevalence of DFUs across the two types of diabetes.^10–13^ Most of the available data derives from studies that have examined temporal trends in DFU occurrence across large population-based cohorts and national registries, providing valuable insight into how the burden of DFUs has evolved over time in T1DM and T2DM (Table 1).

**Table 1.** Summary of key findings from included studies.

Study	Country & study design (with period)	Data source	Population (n)	Outcome	Key findings (T1DM vs T2DM)
Riise et al. (2024)^10^	Norway – Repeated cross-sectional (1995–2019)	Population-based survey (HUNT study)	HUNT2 (1,630); HUNT3 (1,824); HUNT4 (2,393)	DFU prevalence	Higher in T1DM across waves (14.2% → 7.2%) vs T2DM (10.2% → 5.0%)
Rasmussen et al. (2017)^11^	Denmark – Retrospective cohort (2001–2014)	Hospital diabetes clinic + foot clinic registry	T1DM (5,582); T2DM (7,098)	DFU incidence	Lower incidence in T1DM (5.8) vs T2DM (11.3) /1,000 pt-yrs
Roikjer et al. (2022)^12^	UK – Population-based cohort (2007–2017)	Primary care EHR database (CPRD)	129,624	First-ever DFU incidence	T1DM: 1.6 vs T2DM: 2.5 /1,000 pt-yrs
Eckert et al. (2024)^13^	Germany/Austria – Registry-based observational (period Ν/Α)	National diabetes registry (DPV)	T1DM (5,352); T2DM (58,112)	DFU prevalence	T1DM 5.0% vs T2DM 9.5%
Sørensen et al. (2019)^14^	Denmark – Retrospective cohort (1999–2000 & 2011–2012)	Specialist wound healing center	T1DM (65); T2DM (521)	Healing time	No significant difference between T1DM and T2DM
Ince et al. (2007)^15^	UK – Retrospective cohort (period Ν/Α)	Specialist diabetes foot clinic	T1DM (62); T2DM (384)	Healing outcomes	Diabetes type not associated with healing time
Markuson et al. (2009)^16^	USA – Observational cohort (period Ν/Α)	Wound care clinic	63	Healing outcomes	No significant difference between T1DM and T2DM
Oyibo et al. (2001)^17^	UK – Observational cohort (period Ν/Α)	Hospital-based cohort	T1DM (21); T2DM (173)	Healing outcomes	No significant difference in outcomes
Ndosi et al. (2018)^18^	UK & Europe – Prospective multicenter observational study (period Ν/Α)	Multicenter clinical cohort	299	6–12 month outcomes	Outcomes not stratified by diabetes type

T1DM, Type 1 diabetes mellitus; T2DM, Type 2 diabetes mellitus; py, per year; pt-yrs, patient-years; N/A, not available.

Repeated population-based analyses from the Trøndelag Health Study (HUNT) in Norway provide valuable information on temporal trends in DFU burden across diabetes mellitus types.^10^ Across three large cross-sectional surveys, HUNT2 (1995-1997), HUNT3 (2006-2008) and HUNT4 (2017-2019), data on the lifetime prevalence of DFUs were obtained from 1,630, 1,824 and 2393 adults with diabetes, respectively. Among individuals with T1DM, lifetime prevalence declined across survey waves, falling from 14.2% [95% Confidence Intervals (CI): 10.5-18.8] in HUNT2 to 9.0% (95% CI: 5.6-13.8) in HUNT3 and 7.2% (95% CI: 4.8-10.5) in HUNT4. Similarly, in individuals with T2DM, lifetime prevalence of a DFU was 10.2% (95% CI: 8.7-12.0) in HUNT2, decreasing to 7.3% (95% CI: 6.2-8.8) in HUNT3 and 5.0% (95% CI: 4.1-6.0) in HUNT4.^10^ Although the overall burden decreased in both groups, individuals with T1DM consistently demonstrated a higher lifetime prevalence across all survey waves. These findings highlight that differences between T1DM and T2DM persist even in the context of improving population-level risk-factor management, reinforcing the need for dedicated assessment of DFU prevalence by diabetes type.

Long-term incidence trends were also explored in a large observational study from Denmark, which analyzed electronic patient records from a specialized diabetes center with a multidisciplinary foot clinic between 2001 and 2014.^11^ This study included 5,582 individuals with T1DM and 7,098 individuals with T2DM. Over the 14-year period, the incidence of first-ever DFU declined substantially in both diabetes types. Among patients with T1DM, 255 developed a DFU, corresponding to an incidence of 5.8 per 1,000 patient-years (95% CI: 5.1-6.5), with a marked decrease from 8.1 (95% CI: 5.4-11.9) in 2002 to 2.6 (95% CI: 1.3-5.3) in 2014 (p=0.0059). In T2DM, 310 individuals developed a DFU, yielding an incidence of 11.3 per 1,000 patient-years (95% CI: 10.1-12.6), which similarly decreased from 17.0 (95% CI: 12.2-23.8) in 2002 to 8.7 (95% CI: 5.3-14.1) in 2014 (p=0.03). The reduction in incidence in both groups was primarily driven by a decline in neuropathic ulcers, whereas neuro-ischemic and ischemic ulcer rates remained comparatively stable. Despite the overall improvement, incidence remained higher in T2DM.^11^

Data from the United Kingdom (UK) also highlight clear differences in DFU incidence between T1DM and T2DM.^12^ An observational, population-based cohort study using the CPRD GOLD primary care database identified 129,624 individuals with diabetes between 2007 and 2017 and examined first-ever DFUs using validated Read codes, the structured clinical coding system historically employed in UK primary care to record diagnoses and clinical events. Over this 11-year period, the mean incidence rate was higher in T2DM than in T1DM [2.5 per 1,000 person-years (95% CI: 2.1-2.9) versus 1.6 per 1,000 person-years (95% CI: 1.3-1.9), respectively]. Although incidence declined modestly in T2DM over time, no meaningful change was observed in T1DM.^12^ Notably, and in contrast to the Norwegian data, where declines in DFU prevalence and incidence were evident across both diabetes types, the UK study showed reductions exclusively in T2DM, with no significant improvement detected in T1DM.

Additional evidence on differences between T1DM and T2DM comes from a large retrospective analysis of real-world data from the German/Austrian European diabetes registry, which included 63,464 adults (5,352 with T1DM and 58,112 with T2DM) and examined factors associated with DFUs and subsequent amputations.^13^ Ιn this cohort, DFUs were nearly twice as common in T2DM than in T1DM (9.5% vs. 5.0%), and several risk factors differed markedly by diabetes type. Among individuals with T1DM, higher glycated hemoglobin (A1C) levels, elevated triglycerides, and unfavorable low-density lipoprotein) cholesterol (LDL-C)/high-density lipoprotein cholesterol (HDL-C) or total cholesterol/HDL-C ratios were more strongly associated with DFU development when compared with matched controls without DFUs. In contrast, in individuals with T2DM, lifestyle-related factors such as smoking and alcohol abuse, as well as insulin therapy, were more prominent correlates of DFUs, while A1C differences between DFU and non-DFU groups were not significant. Across both diabetes types, male sex, greater height, and the presence of diabetes complications, including neuropathy, peripheral arterial disease (PAD), nephropathy, and retinopathy, were strongly associated with DFU.^13^

Evidence from population-based cohorts, specialized clinical registries, and national databases indicates that the epidemiology and clinical burden of DFUs vary across diabetes types; however, these differences are likely influenced by population structure, comorbidity burden, and healthcare system factors rather than diabetes type per se. The divergent DFU frequencies observed across studies (HUNT: T1DM > T2DM; Danish, UK, and German/Austrian European diabetes registry-based cohorts: T2DM > T1DM) may be explained, at least in part, by differences in population structure, referral pathways, and outcome definitions rather than intrinsic differences between diabetes types. The baseline characteristics of the participants in these respective studies provide a clear pathophysiological rationale for these discrepancies. In the population-based HUNT study, individuals with T1DM exhibited a significantly longer diabetes duration and earlier chronological exposure to chronic hyperglycemia compared to their T2DM counterparts, explaining the higher lifetime cumulative prevalence in the T1DM cohort. Conversely, in the Danish and German/Austrian hospital-based and specialist clinic registries, the T2DM cohorts were characterized by advanced age and a markedly higher prevalence of lifestyle-related risk factors, insulin requirement (as a marker of disease severity), and macrovascular peripheral arterial disease (PAD). Furthermore, specialist clinic registries are inherently prone to referral bias; while nearly all T1DM patients are captured in secondary care, T2DM patients are often only referred to multidisciplinary clinics when they present with severe, neuro-ischemic, or hard-to-heal ulcers, thereby inflating the observed incidence of DFUs in the T2DM registry arms. These observations should be interpreted with caution given the heterogeneity in study design, populations, and case ascertainment methods.

Collectively, these findings suggest that comparisons of DFU frequency between T1DM and T2DM are sensitive to study design, care setting, and case ascertainment methodology, and should therefore be interpreted within the context of underlying comorbidity burden and healthcare structure. In this context, most large observational studies indicate a higher incidence of DFUs in individuals with T2DM than in those with T1DM.^11–13^

Across diverse healthcare settings as well, individuals with T2DM generally exhibit a higher incidence of DFUs than those with T1DM, reflecting the larger population burden and the contribution of comorbidities such as neuropathy, PAD, and lifestyle-related risk factors.^11–13^ In contrast, lifetime prevalence estimates from population surveys suggest that individuals with T1DM may experience a comparatively higher cumulative burden of DFUs, likely reflecting longer diabetes duration and earlier exposure to chronic complications.^10^ Poorer glycemic control and adverse lipid profiles were more prominently correlated with T1DM, whereas earlier diagnosis and intensified multifactorial risk management in T2DM over the study period contributed to a greater reduction in foot ulcer risk in this group.^10^ The basis for interpreting these divergent frequency trends as reflections of underlying clinical phenotypes, rather than intrinsic properties of diabetes type, stems from the explicit baseline and clinical characteristics reported within the primary studies. In the population-based HUNT surveys, individuals with T1DM exhibited a vastly longer median disease duration and earlier exposure to chronic metabolic insults compared to those with T2DM, which directly accounts for their higher lifetime cumulative prevalence. Conversely, in the Danish and German/Austrian registry cohorts, the higher recorded annual incidence in T2DM was strongly associated with an older demographic profile, a significantly higher baseline prevalence of macrovascular complications (PAD), and lifestyle risk factors. Therefore, the descriptive synthesis of these data reveals that diabetes type serves as a clinical proxy for distinct clusters of age, disease duration, and macrovascular burden, which collectively dictate the epidemiological presentation of DFUs across different healthcare structures.

## 4. Ulcer course and healing outcomes

The wound-healing process is impaired at multiple stages of tissue repair in individuals with diabetes, leading to slower and often incomplete resolution of DFUs.^19^ Under optimal circumstances, most DFUs are expected to show substantial improvement within 4 weeks, and a hard-to-heal ulcer is generally defined as one that fails to reduce in size by at least 50% after 4 weeks of appropriate standard care.^20,21^ Factors such as infection and PAD further diminish healing potential and contribute to delayed or incomplete ulcer resolution.^21^ Comparative data on DFU course and healing outcomes between individuals with T1DM and T2DM remain scarce.^14,15^ Importantly, available evidence suggests that ulcer healing is primarily determined by local wound characteristics and systemic factors—particularly perfusion status, infection severity, and comorbidity burden—rather than diabetes type per se.

### 4.1. Healing time

A retrospective study examined DFU healing outcomes over time at the Copenhagen Wound Healing Center, Bispebjerg Hospital, a highly specialized referral center treating individuals with complex and hard-to-heal DFUs.^14^ The analysis compared patients managed during two distinct periods, 1999-2000 and 2011-2012, with the primary aim of assessing differences in healing time across these cohorts. Medical records from all patients with DFUs treated in the specified years were reviewed. Overall, 651 patients were included in the study, contributing a total of 1,048 DFUs. Among them, 80% had T2DM, 10% had T1DM, and 10% were categorized as insulin-dependent diabetes mellitus. In the 1999/2000 sample, patients with T1DM exhibited longer median DFU duration compared with those with T2DM; nevertheless, the difference was not statistically significant (7.9 months vs. 5.8 months; p = 0.3). In the 2011/2012 cohort, a different pattern was observed, with median DFU duration being longer in T2DM than in T1DM (6.7 months vs. 5.5 months), although this difference was also not statistically significant (p = 0.9). It is noteworthy that individuals with T1DM were substantially younger than those with T2DM in both time periods: in 1999/2000, the mean age at ulcer onset was 50 years in T1DM and 67 years in T2DM, and in 2011/2012, approximately 59 years vs. 70 years, respectively. These age differences were statistically significant (p < 0.001).

Patients with T1DM also had markedly longer diabetes duration at DFU onset than those with T2DM (median disease duration 32.7 vs. 12.3 years in 1999/2000 and 36.3 vs. 13.0 years in 2011/2012), supporting diabetes duration as a key explanatory factor underlying differences in DFU burden between T1DM and T2DM.

Another retrospective study examined the association between ulcer healing time and baseline patient and ulcer characteristics within a specialist clinical setting.^15^ All individuals referred to the clinic over a four-year period were included, and detailed information was recorded at presentation. A total of 449 participants were enrolled; 13.9% had T1DM (n = 62) and 86.1% had T2DM (n = 384). PAD was present in 42.7% of participants, and 80% had peripheral neuropathy. The median ulcer duration at presentation was 29 days. Patients were followed for up to one year, with time to healing defined as the primary outcome. In both univariate and multivariate analyses, diabetes type was not associated with healing time, whereas ulcer-related and vascular factors were more relevant determinants of outcome.^15^

Another study examined factors associated with DFU healing in a small cohort.^16^ A total of 63 individuals with DFUs were included, of whom 9 had T1DM and 54 had T2DM. Among the 63 ulcers, 36 healed, 26 did not heal, and healing status could not be determined for one ulcer. Of the 9 individuals with T1DM, 77.8% (7/9) experienced wound healing, compared with 53.7% (32/54) of those with T2DM.^16^ As a p-value was not reported in the original study, we conducted a Pearson chi-square test to compare healing outcomes between T1DM and T2DM. The analysis did not show a statistically significant difference between groups (p = 0.29). Although numerically higher healing rates were observed in T1DM, the absence of statistical significance and the very small sample size limit any meaningful interpretation. Importantly, no adjustment for ulcer severity or vascular status was performed, further restricting conclusions regarding the role of diabetes type. Similarly, in a study including 194 patients, 173 of whom had T2DM (89%), no differences in healing, amputation, or non-healing outcomes were observed between individuals with T1DM and those with T2DM, further supporting the lack of an independent effect of diabetes type on DFU outcomes.^17^

Overall, current evidence suggests that the healing time for DFU does not significantly differ between individuals with T1DM and T2DM, with outcomes primarily driven by ulcer-specific and vascular factors. While some studies have observed minor variations in median ulcer duration, these differences were not statistically significant.

### 4.2. Ulcer recurrence

DFU recurrence remains a major challenge in the long-term management of DFUs, with recurrence rates up to 40% within one year, 60% within three years, and up to 65% within five years.^1^ These high recurrence rates have also been confirmed in cohorts undergoing vascular interventions for chronic limb-threatening ischemia, where recurrence reaches approximately 50–58% within three years and they underscore the chronic and multifactorial nature of DFU, which is primarily driven by underlying vascular disease, neuropathy, and persistent biomechanical risk factors rather than diabetes type per se.^22^

A retrospective cohort study from the Steno Diabetes Center Copenhagen foot clinic evaluated DFU recurrence and development of new lesions in 780 patients with previously healed DFUs.^23^ Among them 643 had T2DM and 137 had T1DM. Individuals with T2DM showed a modestly higher rate of recurrent or new ulceration compared with those with T1DM (rate ratio 0.81; 95% CI: 0.67–0.99). However, this difference was borderline, with the upper confidence interval approaching unity, and should be interpreted with caution, particularly given the imbalance in sample size between T2DM and T1DM. Importantly, this study also demonstrated that ulcer phenotype, particularly the presence of neuro-ischaemic or critically ischaemic ulcers, was a major determinant of progression and recurrence, supporting the concept that vascular status and ulcer characteristics play a more central role than diabetes type itself.^23^

A long-term prospective study followed patients with active DFUs in Germany (n = 222) and the Czech Republic (n = 99) over approximately 15 years. DFU recurrence was common, occurring in 69% (154/222) of patients in Germany and 70% (69/99) in the Czech Republic. Notably, in the Czech cohort, T2DM was associated with a significantly shorter time to recurrence, with a hazard ratio (HR) of 2.57 (95% CI: 1.18–5.62; p = 0.018), indicating that individuals with T2DM had more than twice the risk of earlier ulcer recurrence compared with those without T2DM. Although a significant association between T2DM and shorter time to recurrence was observed in one cohort, this was not consistently replicated across both populations, suggesting heterogeneity in underlying risk structures rather than a uniform effect of diabetes type. In contrast, this association was not statistically significant in the German cohort (HR 1.21; 95% CI: 0.66–2.22; p = 0.538).^24^

Overall, DFU recurrence is highly common in both T1DM and T2DM and represents a major unresolved clinical challenge. Although some studies report higher or earlier recurrence in T2DM, findings are inconsistent and likely reflect differences in age, peripheral arterial disease burden, and ulcer phenotype. Taken together, the available evidence supports the concept that recurrence is primarily driven by persistent underlying neuropathic and vascular pathology, with diabetes type acting as a contextual marker rather than an independent determinant of risk.

## 5. Complications related to diabetic foot ulcers

### 5.1. Amputations

LEA remains one of the most severe complications of DFUs, reflecting advanced infection, ischemia, or failure of ulcer healing.^1^ Herein, we summarize the available evidence on minor and major LEA rates in T1DM and T2DM, highlighting differences in lower-extremity amputation risk across T1DM and T2DM and the extent to which these differences may be explained by underlying clinical phenotypes rather than diabetes type per se. (Table 2).

**Table 2.** Summary of key findings from included studies.

Author (Year)	Study design (with period)	Data source	Population (n)	Outcome	Key findings (T1DM vs T2DM)
Ogurtsova et al. (2021)^24^	Prospective cohort (Germany/Czech Republic, ∼15-year follow-up)	Clinical cohorts (specialist centers)	GER/CZ T1DM: 49/24 T2DM: 272/75	LEA	Higher LEA risk in T2DM vs T1DM (GER: HR 1.21 CZ: HR 2.57)
Deruaz-Luyet et al. (2020)^25^	Retrospective cohort (USA, 2010–2014)	Administrative claims database (IBM MarketScan)	T1DM (120,129) T2DM (1,679,877)	Minor LEA, Toe amputation	Higher minor LEA incidence in T1DM (4.85 vs 1.53 per 1,000 pt-yrs); toe amputations 4.49 vs 1.43
Kurowski et al. (2015)^26^	Population-based retrospective cohort (Australia, 2000–2010)	Linked administrative health data	T1DM (364) T2DM (3,399)	LEA	Incidence higher in T1DM than T2DM (724 vs 564 per 100,000 person-years)
Chamberlain et al. (2022)^27^	National population-based retrospective cohort (Scotland, UK; period N/A)	National diabetes registry	T1DM (23,395) T2DM (210,064)	LEA or death	Increased risk in both: T1DM ×2.09; T2DM ×1.65
Ezzatvar & Garcia-Hermoso (2023)^28^	Systematic review and meta-analysis (global, 2010–2020)	Published studies	(505,390)	Minor/Major LEA	Higher LEA rates in T1DM (148.6/100.8 vs 75.5/40.6 per 100,000)
Roikjer et al. (2020)^29^	Nationwide retrospective cohort (Denmark, 1997–2017)	National administrative registry	(462,743)	Site-specific LEA	Higher absolute incidence and declines in T2DM vs T1DM
Carey et al. (2018)^30^	Matched population-based cohort (UK, up to 2008)	Primary care database (CPRD)	T1DM (5,863) T2DM (96,630)	Infection	Bone/joint infection risk: T1DM ×22.3; T2DM ×4.9; STI: T1DM ×2.8; T2DM ×2.0
Zhang et al. (2025)^31^	Mendelian randomization (international, not time-bound)	GWAS datasets	T1DM (4,320) T2DM (65,085)	OM	Increased OM risk in both T1DM 14–26%; T2DM 22–39%
Malone et al. (2024)^32^	Observational cohort (Australia, period N/A)	Clinical infection database	T1DM (22) T2DM (304)	STI; OM	Majority of infections in T2DM (94%); similar pattern for STI (93%); OM (94%)
Magliano et al. (2015)^33^	Nationwide cohort study (Australia, period N/A)	National registry and mortality data	T1DM 7.7% (85,144) T2DM 92.3% (1,023,838)	Infection-related mortality	Higher relative mortality risk in T1DM (×4.4 vs ×1.5)
Macdonald et al. (2021)^34^	Meta-analysis (global, not time-bound)	Published studies	(16,159)	Microbiology	S. aureus (21–23%); MRSA (∼18%); polymicrobial (∼60%)

T1DM, Type 1 diabetes mellitus; T2DM, Type 2 diabetes mellitus; LEA, lower extremity amputation; OM, osteomyelitis; STI, soft tissue infection; MRSA, methicillin-resistant Staphylococcus aureus; HR, hazard ratio; IRR, incidence rate ratio; OR, odds ratio; pt-yrs, patient-years; GWAS, genome-wide association studies; CPRD, Clinical Practice Research Datalink; GER, Germany; CZ, Czech; USA, United States of America; N/A, Not available.

A large retrospective cohort study using the IBM MarketScan database evaluated the incidence of lower LEAs among individuals with T1DM and T2DM in the United States between 2010 and 2014.^25^ The analysis included 120,129 adults with T1DM and approximately 1.7 million adults with T2DM, each matched to a non-diabetic control by age, sex, and calendar time. Overall, the incidence of LEA was higher in T1DM than in T2DM, with minor amputations, mainly toe amputations, being the most common in both groups. The incidence rates of minor LEA were 4.85 per 1,000 patient-years in T1DM compared with 1.53 per 1,000 patient-years in T2DM, with corresponding toe amputation rates of 4.49 and 1.43 per 1,000 patient-years, respectively.^25^ These differences reflect higher crude incidence rates of LEA observed in T1DM compared with T2DM, largely reflecting differences in population structure and cumulative exposure to diabetes-related complications.

A population-based study from Western Australia examined temporal trends in LEAs among adults with diabetes between 2000 and 2010. Using linked health data, the authors identified 5,891 non-traumatic amputations.^26^ Of these, 364 occurred in individuals with T1DM, 3399 in people with T2DM, and 2128 in individuals with cardiovascular disease but without diabetes. The average annual rates of total LEAs were highest in T1DM at 724 per 100,000 person-years, followed by T2DM at 564 per 100,000 person-years, and were substantially lower in individuals with cardiovascular disease without diabetes (66 per 100,000 person-years). Over time, total LEA rates declined in T1DM from 1,028 to 795 per 100,000 person-years, although this reduction was not statistically significant. Similar downward trends were seen in T2DM. Initial major LEAs in T2DM fell from 111.1 to 60.5 per 100,000 person-years, corresponding to an annual decrease of −6.6% vs. −8.2% in T1DM. Downward trends in initial minor LEAs were observed in the T1DM group (−1.7% vs −1.4% in T2DM), but these changes were not statistically significant. Furthermore, among individuals with T1DM, recurrent LEAs rates remained largely unchanged throughout the study period. In contrast, recurrent LEAs were substantially more frequent in individuals with T2DM, with an average annual age- and sex-standardized rate of 35,440 per 100,000 person-years. Notably, recurrent minor LEAs increased significantly among individuals with T2DM, rising by +3.5% annually (95% CI: +1.3 to +5.7).^26^ These findings emphasize that while initial LEAs rates have improved, particularly in T2DM, recurrent LEAs remain a major unresolved challenge.

Further reinforcing this pattern, a national Scottish retrospective study of 233,459 adults with diabetes (23,395 with T1DM and 210,064 with T2DM) demonstrated substantial differences in LEA outcomes by diabetes type.^27^ In total there were 13,093 (5.6%) people who had a previous foot ulceration, 9,023 people who developed a first ulcer, 48,995 who died, and 2,866 who underwent minor or major LEAs during follow-up. Among individuals with a prior DFU, 33,9% of T1DM vs 16,9% of T2DM (261 of 2,105 T1DM and 967 of 10,988 T2DM) underwent an amputation, compared with 224 of 21,290 (12.9%) and 1,414 of 199,076 (3.3%), respectively, among those without an ulcer history. Stratifying by amputation type, in T1DM with prior DFU, 145 were major and 156 were minor amputations, whereas in T2DM with prior DFU, 538 were major and 579 were minor. For individuals without prior DFU, T1DM had 110 major and 137 minor, and T2DM had 748 major and 831 minor amputations. After adjustment for key risk factors, the hazard of the composite outcome “amputation or death” associated with previous ulceration remained higher in T1DM than in T2DM (adjusted HR 2.09; 95% CI: 1.89–2.31 vs. 1.65; 95% CI: 1.60–1.70; p < 0.001).^27^ This pronounced hazard in T1DM cohorts must also be contextualized within the framework of survival bias and competing risks; individuals with T1DM develop diabetes at a much younger age, meaning that by the time they present with a DFU, they frequently possess decades of cumulative metabolic insult and severe microvascular changes, whereas older individuals with T2DM face competing risks from macrovascular mortality before reaching similar thresholds of long-term tissue damage. These findings likely reflect the higher burden of long-standing neuropathy and peripheral arterial disease in individuals with T1DM who develop foot ulceration, as well as longer cumulative exposure to diabetes-related complications rather than an intrinsic effect of diabetes type.

Additional evidence comes from a large cohort study which included 10,156 individuals with diabetes (34% with T1DM and 66% with T2DM) and PAD who were followed for a median of 34 months. During follow-up, 23% experienced a limb event (critical limb ischaemia or vascular amputation) and 12% experienced a cardiovascular event (myocardial infarction or ischemic stroke). Individuals with T1DM had a significantly higher incidence of limb events than those with T2DM. After adjustment for age, hypertension, hyperlipidemia, smoking, A1C, and other clinical factors, T1DM remained associated with a higher risk of limb events, with an adjusted HR of 1.58 (95% CI: 1.44–1.73; p < 0.0001). In contrast, rates of cardiovascular events (myocardial infarction and ischemic stroke) and all-cause mortality were similar between T1DM and T2DM.^35^

A meta-analysis published in 2023 that included several of the aforementioned studies provided global estimates of diabetes-related amputation incidence between 2010 and 2020.^28^ Across 23 observational studies encompassing 505,390 diabetes-related LEAs worldwide, the analysis confirmed that both minor and major LEAs incidence rates are substantially higher in individuals with T1DM compared with those with T2DM. For minor amputations, the pooled incidence rate was 148.6 per 100,000 (95% CI: 65.0-339.7) in T1DM vs. 75.5 per 100,000 (95% CI: 29.9-190.5) in T2DM. For major amputations, the incidence rate was 100.8 per 100,000 (95% CI: 53.7-189.0) in T1DM compared with 40.6 per 100,000 (95% CI: 11.0–149.3) in T2DM.

In contrast to this pattern, a large European registry study including 63,464 adults with diabetes (5,352 with T1DM and 58,112 with T2DM) evaluated LEA outcomes among patients with diabetic foot ulcers.^13^ In this cohort, amputation occurred in 36.5% of individuals with T1DM and 38.1% of those with T2DM. After adjustment for relevant clinical factors, the odds of LEA were significantly higher in T2DM compared with T1DM (adjusted odds ratio [aOR] 1.17; 95% CI: 1.06–1.30; p = 0.003).^13^ This likely reflects differences in age distribution and a higher burden of peripheral arterial disease and multimorbidity among individuals with T2DM undergoing foot ulcer care.^13^

In addition, a comprehensive Danish nationwide study spanning two decades (1997-2017, total 462,743 people with T1DM or T2DM and 1.39 million without diabetes) demonstrated that absolute LEA incidence was consistently higher in T2D across all amputation types.^29^ Specifically, for trans-femoral amputations, incidence rates declined from 0.18 to 0.12 per 1000 person-years in T1DM and from 0.25 to 0.18 per 1000 person-years in T2DM, corresponding to annual absolute reductions of −0.022 and −0.032, respectively. Trans-tibial amputations fell from 0.36 to 0.16 per 1000 person-years in T1DM and from 0.50 to 0.21 per 1000 person-years in T2DM, reflecting steeper annual declines in both groups (−0.072 in T1DM versus −0.090 in T2DM). Similarly, below-ankle amputations decreased from 0.28 to 0.13 per 1000 person-years in T1DM and from 0.45 to 0.18 per 1000 person-years in T2DM. Across all amputation levels, patients with T2DM consistently had higher incidence rates than those with T1DM, though both groups showed substantial reductions over the two decades.^29^

Overall, the evidence indicates that the risk of LEAs is elevated in both T1DM and T2DM, but the relative contribution of each diabetes type varies across populations and study designs. Large administrative and population-based cohorts frequently report higher incidence rates of both minor and major amputations in individuals with T1DM, a pattern generally supported by meta-analytic estimates. Some of these studies reported baseline differences between diabetes types that may be relevant when interpreting these findings. For example, in the Scottish national registry, individuals with T1DM and a history of DFU presented with a significantly younger age but a markedly longer duration of diabetes compared to those with T2DM, indicating that earlier onset and longer cumulative exposure drive this elevated risk. In contrast, real-world clinical registries and DFU-specific cohorts suggest that among individuals with established foot ulcers, amputation risk may be similar or higher in T2DM. This pattern is typically explained by the baseline case-mix of these cohorts, such as the German/Austrian DPV registry, where patients with T2DM and DFUs were significantly older and presented with a substantially higher baseline prevalence of peripheral arterial disease (PAD) and multi-morbidity than their T1DM counterparts. These discrepancies should be interpreted cautiously, as most of the available studies were not specifically designed to determine whether observed differences in amputation risk are attributable to diabetes type itself or to associated clinical characteristics. 

Consequently, the current literature does not allow definitive conclusions regarding the independent contribution of diabetes type to amputation risk, suggesting that diabetes type acts as a contextual marker of differing clinical phenotypes rather than an independent prognostic driver.

### 5.2. Infections

Once an ulcer develops, infection is common and roughly 50–60% of ulcers become infected.^1,36^ Despite the clinical importance of DFU-associated infections, including soft-tissue infections (STIs), cellulitis, abscess formation, and osteomyelitis (ΟΜ), evidence directly comparing infectious outcomes between individuals with T1DM and T2DM in DFU populations remains limited, and much of the available literature derives from broader diabetes cohorts rather than DFU-specific studies. Few studies stratify infection prevalence, microbial profiles, resistance patterns, or infection-related healing outcomes by diabetes type, leaving uncertainty regarding whether susceptibility to infection or infection severity differs meaningfully between T1DM and T2DM.^37,38^

A large population-based retrospective cohort study compared 102,493 English primary care patients aged 40-89 years with diagnosed diabetes by 2008 (5,863 with T1DM and 96,630 with T2DM) with 203,518 age-, sex-, and practice-matched individuals without diabetes.^30^ Marked differences in infection risk were observed between diabetes types. Among individuals with T1DM, the incidence rate ratio (IRR) for bone and joint infections, predominantly OM, was 22.34 (95% CI: 12.12–41.20), compared with 4.93 (95% CI: 4.34–5.61) in those with T2DM. A similar pattern was seen for STIs: cellulitis carried a higher excess risk in T1DM (IRR 2.84; 95% CI: 2.48–3.25) than in T2DM (IRR 2.03; 95% CI: 1.97–2.08), and “other skin infections” followed the same gradient (T1DM IRR 2.15; 95% CI: 1.96–2.37 vs. T2DM IRR 1.72; 95% CI: 1.67–1.78). Although T2DM accounts for most absolute infection cases due to its far greater population prevalence and older, more comorbid demographic profile, these findings suggest a higher relative risk of deep and severe infections in individuals with T1DM; however, this should be interpreted in the context of differences in age, disease duration, and comorbidity burden between diabetes types. Importantly, this study evaluated infection risk across a general diabetes population and did not specifically include patients with diabetic foot ulcers or assess DFU-related infectious complications such as foot infection severity or osteomyelitis in the context of ulcer disease. Therefore, while it supports an increased susceptibility to infections in T1DM, it does not directly inform DFU-specific infection outcomes or foot-related infectious pathology.

Moreover, a two-sample Mendelian randomization study using genome-wide association data from more than 1 million participants, demonstrated that both forms of diabetes causally increase OM risk, with OR of 1.14-1.26 for T1DM and 1.22-1.39 for T2DM.^31^ The causal relationship persisted when diabetes with complications was analyzed separately (T1DM and complications: OR 1.26; T2DM and complications: OR 1.34). Mediation analyses identified A1C and body mass index (BMI) as significant factors of this effect, as A1C mediated roughly 5% of the diabetes-OM link in T1DΜ and 28% in T2DΜ, while BMI contributed an additional 3% in T1DM and 4% in T2DM. These findings indicate that poor glycemic control and obesity potentiate the risk of bone infection in both types of diabetes. These findings indicate a causal relationship between diabetes and osteomyelitis risk at the population level; however, they do not differentiate between infection sources or specifically isolate diabetic foot ulcer-related osteomyelitis.

In line with these findings, in a large Australian cohort which analyzed 647 diabetic foot infections (DFIs) in 397 patients, the majority of infections occurred in individuals with T2DM (94%), while only 6% were in T1DM.^32^ Although direct statistical comparisons between types of diabetes were not performed due to this imbalance, the data clearly reflect the predominance of DFIs in T2DM, consistent with the higher burden of PAD, neuropathy, and comorbidities in this group. The study distinguished skin and soft-tissue infections (SSTIs) from OM, finding similar distributions of diabetes type across both infection categories (T1DM: 7% in SSTIs vs. 93% T2DM, 6% vs 94% in OM respectively). Importantly, PAD and ischemia grades, major determinants of infection outcome, were more prevalent and severe in T2DM, whereas T1DΜ patients tended to present with neuropathic ulcers and less macrovascular compromise. Infection resolution rates were 82% for SSTIs and 56% for OM, with treatment failure strongly linked to PAD and higher ischemia grades, rather than diabetes type per se. These findings reinforce that diabetic foot infection outcomes are primarily driven by vascular status and ulcer severity rather than intrinsic differences between diabetes types.

In addition, significant excess mortality risk from ΟΜ among individuals with diabetes was reported in a nationwide Australian cohort of over one million patients.^33^ The standardized mortality ratio for ΟΜ reached 29.6 (95% CI 14.7–59.1) in Τ1DM, compared with 3.3 (2.8–3.9) in T2DM, indicating a higher relative mortality burden in T1DM; however, this likely reflects differences in disease duration and complication burden rather than a direct effect of diabetes type. The authors noted that over 60% of ΟΜ deaths were accompanied by sepsis and an additional 13% by pneumonia, highlighting that diabetic foot–related bone infection often represents the terminal event in systemic infection. These results state that ΟΜ constitutes one of the most severe and fatal infection types in diabetes, with the relative burden particularly pronounced in T1DM patients. However, these estimates reflect osteomyelitis-related mortality in a broad diabetes population and are not restricted to diabetic foot ulcer-related infections; therefore, they should be interpreted as markers of overall infection vulnerability rather than DFU-specific mortality risk.

Although most studies do not stratify microbiological findings by diabetes type, the global meta-analysis which pooled data from 112 studies and 16,159 patients, provides the most comprehensive overview of DFU microbiology.^34^ Across both T1DM and T2DM populations, Staphylococcus aureus (S. aureus) was the most frequently isolated pathogen (21–23% of isolates). Other common organisms included Pseudomonas spp., Escherichia coli, Enterococcus spp., and Proteus spp., with polymicrobial growth observed in nearly 60% of cultures. The study revealed that the balance between Gram-positive and Gram-negative flora correlates more with clinical and socioeconomic context than diabetes type. Isolates from high-income settings were predominantly Gram-positive, while Gram-negative species dominated in low- and middle-income regions. Overall, both T1DM and T2DM share a broadly similar ulcer microbiology, dominated by S. aureus and opportunistic Gram-negative bacilli, but vary in infection complexity according to disease chronicity, ischemia, and prior antibiotic exposure rather than underlying diabetes type.

In accordance with the above, a multicenter prospective study of 299 participants (13.4% with T1DM and 86.6% with T2DM) with clinically infected DFUs assessed clinical, microbiological, and healing outcomes over a 12-month period.^18^ Although microbiological patterns were broadly similar between groups, type of diabetes was not significantly associated with ulcer healing in either univariable or multivariable analyses. Instead, outcomes were driven by ulcer-specific and vascular factors, notably poor perfusion, as assessed by the PEDIS classification system (Perfusion, Extent, Depth, Infection, Sensation; PEDIS ≥ 2), multiple ulcers, and ulcer duration > 2 months, which strongly predicted non-healing (HR 0.37 and 0.55, respectively). Among microbiological variables, methicillin resistant Staphylococcus aureus (MRSA) isolation correlated with lower healing rates (HR 0.50, p = 0.04), whereas the presence of coagulase-negative staphylococci was associated with improved outcomes (HR 1.53, p = 0.06).

Concerning STIs in a prospective population-based cohort study, both T1DM and T2DM were associated with a significantly increased risk of skin infections compared to non-diabetic controls.^39^ After adjustment for age, sex, and comorbidities, the risk of bacterial skin infections was slightly higher in T1DM (aOR = 1.59, 95 % CI 1.12–2.24) than in T2DM (aOR = 1.33, 95 % CI 1.15–1.54). Notably, recurrent bacterial infections (≥2 episodes per year) were almost threefold more frequent in T1DM (aOR = 2.91) compared with controls, whereas recurrence in T2DM was modest (aOR = 1.36). These findings indicate that while both diabetes types predispose to bacterial skin infections, T1DM carries a higher relative risk, possibly related to higher Staphylococcus aureus carriage among insulin-dependent individuals, whereas T2DM accounts for most cases due to its greater population prevalence.

Despite the distinct immunologic and metabolic backgrounds of T1DM and T2DM, the therapeutic approach to DFIs remains fundamentally the same. Management is dictated by infection severity, ulcer depth, vascular status, and microbiology, not by the underlying diabetes type. Both forms require a multidisciplinary strategy integrating prompt surgical debridement, targeted antibiotic therapy, optimal off-loading, and vascular assessment with revascularization when indicated, as outlined in the latest IWGDF/IDSA guidelines.^40^ According to these recommendations, virulence factors such as biofilm formation, tissue hypoxia, and local mechanical stress operate uniformly across both diabetes types. Consequently, targeted antibiotic selection based on deep tissue cultures and the mandatory implementation of non-removable offloading devices remain the absolute cornerstones of therapy, independent of the metabolic pathway of origin. Glycemic optimization, wound care, and infection control are equally critical in both T1DM and T2DM. Although T1DM and T2DM differ in immune and vascular pathology, these distinctions have not yet translated into type-specific therapeutic protocols. Overall, current evidence does not support diabetes type as an independent determinant of infection outcomes in DFUs, with clinical management appropriately guided by infection severity, vascular status, and microbiological findings. Accordingly, diabetes type should be interpreted as a marker of underlying clinical phenotype rather than a driver of infection management strategy or outcome prediction. These distinctions have not translated into diabetes type–specific therapeutic protocols in clinical practice.

### 5.3. Limitations of the available evidence and methodological considerations

The interpretation of findings in this review is limited by substantial methodological heterogeneity across the included studies. Most studies are observational in design, including retrospective cohorts and registry-based analyses, with relatively few prospective datasets and no randomized controlled trials. As a result, the level of evidence varies considerably, ranging from descriptive epidemiological reports to adjusted multivariable analyses. In addition, there is marked variability in study populations and healthcare settings, including population-based cohorts, specialist foot clinics, and administrative databases, which limits direct comparability between studies. Definitions of key outcomes, particularly diabetic foot ulcer healing, also differ substantially across studies, ranging from complete epithelialization confirmed at follow-up visits to percentage reduction in wound size or clinician-assessed healing. These differences in study design, outcome definitions, and analytical approaches should be considered when interpreting differences between T1DM and T2DM, as they may contribute to the observed heterogeneity in reported outcomes.

Most importantly, a critical limitation is that the primary literature was not methodologically structured or statistically powered to investigate diabetes type as an independent driver versus a covariate of underlying clinical phenotypes. Consequently, many reported similarities or statistically non-significant findings—particularly in smaller clinical cohorts—may suffer from type II errors due to underpowered subgroup analyses. In accordance with methodological principles, this lack of evidence for distinct, type-specific outcomes should not be misinterpreted as definitive proof of clinical equivalence, but rather as an indication of the limitations inherent to historical, type-agnostic data tracking.

## 6. Conclusion

Importantly, once a DFU has developed, current data generally indicate that ulcer course, including healing time and non-healing rates, does not substantially differ between T1DM and T2DM. Instead, outcomes appear to be primarily determined by ulcer-related factors, including perfusion status, infection severity, ulcer duration, and overall disease burden. Differences observed between T1DM- and T2DM-associated DFUs may therefore be more appropriately interpreted as reflecting distinct clinical phenotypes rather than a direct effect of diabetes type itself. Individuals with T2DM more frequently present with neuro-ischaemic ulcers and a higher burden of peripheral arterial disease, which may contribute to increased recurrence risk, whereas T1DM is often characterized by longer diabetes duration and a predominantly neuropathic profile.

Although some studies suggest differences in infection risk or severity, direct comparative evidence in DFU populations remains limited, and microbiological patterns appear broadly similar across diabetes types, being driven primarily by ulcer characteristics and local factors. Importantly, the available evidence includes both direct comparative studies in DFU cohorts and broader diabetes populations providing contextual insights, and should therefore be interpreted with caution. Furthermore, many of the included studies were not specifically designed to distinguish the independent effect of diabetes type from differences in age, diabetes duration, vascular burden, neuropathy, and other comorbidity-related factors.

Overall, diabetes type may not act as an independent determinant of DFU prognosis but rather as a marker of differing clinical and pathophysiological contexts that shape ulcer presentation and downstream outcomes. Current management remains appropriately guided by ulcer severity and vascular status rather than diabetes subtype.

To definitively determine whether diabetes type independently drives DFU risk and disease course, or whether outcomes are entirely dictated by the underlying clinical phenotype, future research must shift away from simple retrospective comparisons. Ideal study designs should utilize large-scale, prospective inception cohorts enrolling patients either at the time of diabetes diagnosis or at the precise onset of their first DFU. From an analytical perspective, standard multivariable regression is often insufficient due to severe confounding by baseline variables like age and disease duration.

Future research should focus on prospective longitudinal cohorts with standardized DFU definitions and detailed characterization of factors such as diabetes duration, glycemic control, neuropathy, peripheral arterial disease, ulcer phenotype, and comorbidity burden. Analytical approaches including propensity-score matching, mediation analyses, and other causal inference methods may help distinguish the independent contribution of diabetes type from that of associated clinical characteristics. Such studies could clarify whether differences in DFU risk, healing, recurrence, and complications are driven by diabetes type itself or by the distinct clinical phenotypes that frequently accompany T1DM and T2DM.

## Data Availability

Data sharing is not applicable to this article as no new data were created or analyzed in this narrative review.*
